# Impact of prior stroke on major clinical outcome in chronic kidney disease: the Salford kidney cohort study

**DOI:** 10.1186/s12882-019-1614-5

**Published:** 2019-11-27

**Authors:** James Tollitt, Aghogho Odudu, Emma Flanagan, Rajkumar Chinnadurai, Craig Smith, Philip A. Kalra

**Affiliations:** 1 Renal Department, Salford Royal NHS Foundation Trust, Stott Lane, Salford, M6 8HD UK; 2 Institute of Cardiovascular Sciences, University of Manchester, Oxford Road, Manchester, UK; 3 Informatics Department, Salford Royal NHS Trust, Salford, UK; 4 Stroke department, Salford Royal NHS Trust, Salford, UK

**Keywords:** Stroke, CKD, Dialysis, Mortality

## Abstract

**Background:**

Chronic kidney disease (CKD) is an independent risk factor for stroke in the general population. The impact of prior stroke on major clinical outcomes in CKD populations is poorly characterised.

**Methods:**

The Salford Kidney Study is a UK prospective cohort of more than 3000 patients recruited since 2002 and followed until March 2018. Multivariable Cox regression examined associations of stroke at two time points; cohort inception, and at dialysis initiation, with risks of death, non-fatal cardiovascular events (NFCVE) and end stage renal disease (ESRD).

**Results:**

277 (9.1%) of 3060 patients suffered a prior stroke and this was associated with mortality, ESRD and future NFCVE after cardiovascular risk factor adjustments. Median survival for prior stroke patients was 40 months vs 77 months in patients without a stroke. Prior stroke was independently associated with mortality (HR 1.20 95%CI 1.0–1.43, *p* = 0.05). Of 579 patients who reached ESRD and commenced dialysis, a prior stroke (*N* = 48) was independently associated with mortality. Median survival for the prior stroke group was 29 months compared with 50 months for the non-stroke group. Only 70 and 75% of patients who had suffered an ischaemic stroke were prescribed antiplatelets or statins respectively.

**Conclusions:**

A diagnosis of stroke is strongly and independently associated with several adverse clinical outcomes for patients with CKD. Prior stroke profoundly alters cardiovascular risk in CKD patients. Greater attention to primary and secondary preventive strategies is warranted which may improve these outcomes.

## Background

Stroke and chronic kidney disease (CKD) are major world health concerns but their interaction is rarely considered. Cerebrovascular disease is the third leading cause of death in the UK [1]. Due to ageing populations and improved survival after stroke [2], more patients are living with these two comorbidities. Patients with CKD are more likely to suffer severe and recurrent strokes [3, 4]. For every 10 mL/min/1.73m^2^ reduction in glomerular filtration rate (GFR), the risk of stroke increases by 7% [5]. Dialysis patients also have an elevated risk of stroke with a much higher mortality [6, 7]. CKD and stroke have shared cardiovascular risk factors. Similarities exist between the brain and the kidney in terms of vascular anatomy, vasoregulatory and bidirectional humoral and non-humoral pathways [8]. Despite this, the increased risk of stroke is not fully explained by aggregation of these traditional risk factors within the CKD population [9]. CKD itself is therefore an independent risk factor for stroke in studies of the general population [9, 10].

The impact of a historical stroke and the development or progression of subsequent CKD has not previously been well characterised. The aims of this study were twofold. Firstly, to investigate the associations between a prior stroke and major cardiovascular and kidney disease outcomes, including non-fatal cardiovascular event (NFCVE), ESRD and death, in a large UK cohort of patients with ND-CKD. Secondly, to determine the impact of history of prior stroke on mortality for patients who were initiated on dialysis.

## Methods

The study used data from the Salford Kidney Study (SKS), previously known as the Chronic Renal Insufficiency Standards Implementation Study (CRISIS), a longitudinal epidemiological cohort study of more than 3000 adults with all-cause ND-CKD recruited since October 2002 [11, 12]. Ethical approval was granted by the regional ethics committee (REC15/NW/0818). Inclusion criteria were patients 18 years or older, referred to a tertiary renal centre (catchment population 1.55million) with an eGFR < 60 mL/min/1.73m^2^ and not requiring immediate renal replacement therapy. Demographic, comorbidity and laboratory data were recorded at baseline and annually. Mortality data were obtained by cross referencing with national mortality data and locally held death certification. Self-reported cerebrovascular and cardiovascular events and event dates were validated following review of clinical records, radiology reports, general practice records, clinical coding and outpatient clinic letters.

**Fig. 1:**
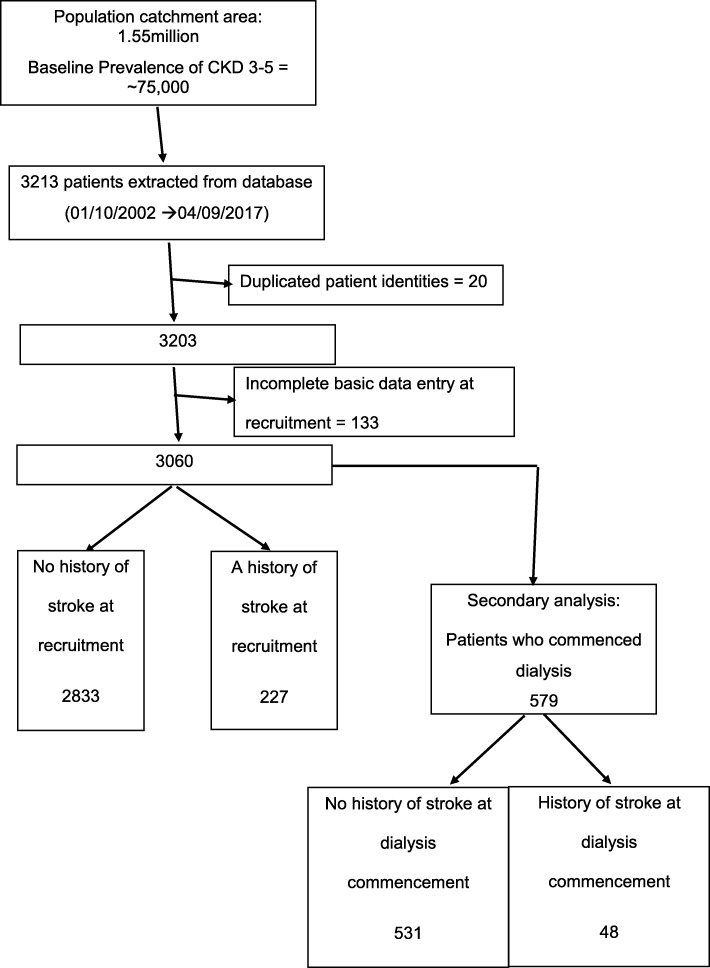
Flow chart for the inclusion of patients within this study

Inclusion criteria for the current analyses are summarised in Fig. 1. Data were analysed on the basis of whether the participant had suffered a stroke prior to recruitment or not. Transient ischaemic attacks (TIAs) were not included in the stroke group because the study set out to specifically ascertain the impact of stroke and not wider cerebrovascular disease on outcomes in CKD. TIA was also difficult to retrospectively prove prior to study commencement because the diagnosis is often clinical and up to 60% of patients referred to TIA clinics do not have a TIA [13, 14]. Hypertension was determined by antihypertensive prescription at recruitment or if patients had a coded diagnosis of hypertension in GP or hospital records. Blood pressure was the mean of 2 stable readings on the first study visit, using an automated sphygmomanometer with an appropriately sized cuff, after at least 5 min of seated rest. Heart failure was classified by the New York Heart Association classification. A composite outcome of NFCVE comprised myocardial infarction, coronary revascularization (including bypass grafting and non-fatal cardiac arrest), cerebrovascular events (stroke or TIA) and newly diagnosed peripheral vascular disease including amputation. Patients were followed from study recruitment until death, commencement of renal replacement therapy or eGFR < 10 mL/min/1.73m^2^ using CKD-EPI formula [15]. For patients not reaching study end points, data were censored on the last hospital visit or on 2^nd^ March 2018. Date of renal replacement commencement and cardiovascular end points were confirmed by a study coordinator who was blinded to the baseline characteristics.

For the analysis of prevalent stroke at time of dialysis commencement, data were analysed using the date of first outpatient dialysis (haemodialysis) or first dialysis exchange in the community (peritoneal dialysis) as study start date. Stroke prior to dialysis commencement consisted of stroke events prior to recruitment and strokes events which occurred during study follow up. Patients were followed until death, transfer out of area or 2^nd^ March 2018.

### Statistical analysis

Univariate analyses were performed stratified by the presence or absence of prior stroke at recruitment. Continuous variables are presented as median (interquartile range) and categorical variables as number (percentage). Between group comparisons were made using Mann-U Whitney for continuous variables and the chi square test for categorical variables. Unadjusted survival was assessed using Kaplan-Meier analysis and significance was assessed using a log rank test. Adjusted survival analysis was performed using multivariable cox regression for the end points of death, ESRD and NFCVE. In order to account for competing risks, hazard ratios were derived by censoring at the competing event [16]. Variables included in the models were selected a priori on the basis of factors known in the literature to be associated with poor outcomes even if they did not have a significant association with a clinical outcome in univariate analysis. Smoking was added last in the models due to a larger proportion of missing data for this variable. Interaction analysis between model variables was also performed. To avoid biased conclusions due to missing data, multiple imputations were also performed (5 iterations) for all variables used in regression models. All hazard ratios are presented alongside 95% confidence intervals. A *p* value of ≤0.05 was considered statistically significant for all analyses. Analyses were performed using SPSS version 23.0.

## Results

### Prevalent stroke at study recruitment

Of 3060 study recruits, 277 suffered a stroke prior to recruitment (9.1%). The median age of the cohort was 67 years with 38 months median follow up. The study comprised of 149,091 patient follow up months. During follow up, the unadjusted stroke incidence was 10 per 1000 patient years. The prevalent stroke group was made up of 158 (69.6%) with ischaemic aetiology, 15 haemorrhagic (6.6%) and 4 having had 2 strokes prior to recruitment, 1 ischaemic and 1 haemorrhagic (1.8%). In 50 of the 277 prior strokes, a haemorrhagic or ischaemic aetiology could not be verified. None of these patients had available radiology. Where stroke aetiology was available, 89% of strokes were ischaemic in nature which is proportionally similar to general population statistics [17].

Baseline characteristics stratified by prior stroke status are summarised in Table 1. In a predominantly Caucasian population those with prior stroke had a greater prevalence of atherosclerotic risk factors including older age (70 vs 60 years), male gender (71% vs 62%), smoking history (80% v 69%), diabetic nephropathy (22% v 16%), renovascular or hypertensive renal disease (46% v 29%), myocardial infarction (26% v 15%), peripheral vascular disease (25% v 13%), atrial fibrillation (14% v 6%) and heart failure (29% v 19%) all *p* < 0.01. Patients with a prior stroke had a lower eGFR (25.2 v 29.5 mL/min/1.73 m^2^, *p* < 0.01) but there was no difference in degree of proteinuria (33.9 g/mol v 34.8 g/mol, *p* = 0.401). Use of antiplatelet agents (69.8% v 40.1% *p* < 0.01) and statin therapy (77.3% v 59.3% *p* < 0.01) were greater in those with a prior stroke but use of renin angiotensin system (RAS) blockers was similar (60.4% v 62.5% *p* = 0.53). Medication prescriptions split by type of stroke are displayed in Additional file 1: Table S1.

**Table 1:** A comparison of baseline characteristics between patients with a history of stroke at recruitment and those without

	Stroke at recruitment		
	No *N* = 2833	Yes *N* = 227	*p*-value (stroke at recruitment v no stroke at recruitment)
	Count (Column%)	Count (Column%)	
Age (years)	66 (54–75)	70 (65–77)	0.000
Male Gender	1750 (61.8%)	162 (71.4%)	0.004
Hemiplegic		65 (28.6%)	
Living alone	571 (20.2%)	45 (19.8%)	0.882
Widowed	399 (14.1%)	39 (17.2%)	0.302
Ethnic group			
Caucasian	2717 (95.9%)	222 (97.8%)	0.376
Non-Caucasian	116 (4.1%)	5 (2.2%)	
Aetiology of Renal Disease			
Renovascular Disease/Hypertension	811 (28.6%)	105 (46.3%)	0.000
Diabetic kidney disease	455 (16.1%)	49 (21.6%)	0.031
Glomerulonephritis/Vasculitis	467 (16.5%)	19 (8.4%)	0.001
Pyelonephritis	154 (5.4%)	6 (2.6%)	0.069
Autosomal dominant Polycystic Kidney Disease	136 (4.8%)	11 (4.8%)	0.976
Other/Unknown	810 (28.6%)	37 (16.3%)	0.000
Smoking history^a^	1832 (69.3%)	169 (80.1%)	0.001
Diabetes	912 (32.2%)	101 (44.5%)	0.000
Systolic Blood pressure (mmHg)^b^	139 (124–154)	142 (129–155)	0.737
Diastolic Blood Pressure (mmHg)^b^	73 (65–80)	71 (62–80)	0.091
Hypertension^c^	2541 (90.2%)	217 (95.6%)	0.004
Myocardial infarction	432 (15.2%)	60 (26.4%)	0.000
Heart failure^d^	516 (18.9%)	64 (29.1%)	0.000
Peripheral vascular disease	377 (13.3%)	56 (24.7%)	0.000
Atrial fibrillation	178 (6.3%)	32 (14.1%)	0.000
Medications			
Antiplatelet^e^	1113 (40.1%)	157 (69.8%)	0.000
Dual antiplatelet^e^	66 (2.4%)	20 (8.9%)	0.000
Anticoagulation^e^	242 (8.7%)	29 (12.9%)	0.036
Antiplatelet and Anticoagulant^e^	35 (1.3%)	6 (2.7%)	0.081
Statin^e^	1644 (59.3%)	174 (77.3%)	0.000
RAS blockade^e^	1735 (62.5%)	136 (60.4%)	0.532
eGFR (mL/min/1.73m^2^)	29.5 (19.2–42.2)	25.2 (16.6–35.5)	0.000
Haemoglobin (g/l)^f^	123.0 (112.0–135.0)	122.0 (111.0–133.0)	0.423
Ferritin (ug/l)^g^	107.0 (52.0–207.0)	108 (46.0–222.0)	0.824
Folate (ug/l)^h^	7.6 (5.7–10.2)	8.0 (5.9–9.2)	0.600
Vitamin B12 (ng/L)^i^	418 (311–563.0)	414.5 (351–486.0)	0.895
Albumin (g/l)^j^	43 (40–45)	42 (39–44)	0.002
Corrected Calcium (mmol/l)^k^	2.30 (2.21–2.39)	2.30 (2.21–2.39)	0.501
Phosphate (mmol/l)^l^	1.12 (0.98–1.28)	1.12 (0.98–1.30)	0.914
Parathyroid Hormone (ng/l)^m^	63.5 (37.0–111.0)	64.0 (43.0–123.0)	0.147
Total Cholesterol (mmol/l)^n^	4.5 (3.7–5.3)	4.2 (3.7–5.0)	0.005
HDL Cholesterol (mmol/l)^o^	1.3 (1.1–1.6)	1.2 (1.0–1.5)	0.001
LDL Cholesterol (mmol/l)^p^	2.1 (1.6–2.8)	2.2 (1.7–2.9)	0.652
Triglycerides (mmol/l)^q^	1.5 (1.0–2.3)	1.5 (1.1–2.4)	0.649
Bicarbonate (mmol/l)^r^	23.5 (20.9–26.1)	23.0 (18.8–24.5)	0.091
C Reactive Protein (mg/l)^s^	3.8 (1.7–8.3)	4.3 (2.1–9.7)	0.091
Urine Protein Creatinine Ratio (g/mol)^t^	33.9 (13.7–113.3)	34.8 (15.0–111.1)	0.401

Continuous variables expressed as median (interquartile range) and categorical variables presented as number (%). eGFR calculated using CKD-EPI formula

*Abbreviations*: *BP* Blood pressure, *RAS blockade* Renin angiotensin blockade, *HDL* High density lipoprotein, *LDL* Low density lipoprotein

Missing data: ^a^204, ^b^475, ^c^18, ^d^115, ^e^62, ^f^183, ^g^385, ^h^2459, ^i^2425, ^j^169, ^k^182, ^l^194, ^m^672, ^n^275, ^o^2764, ^p^280, ^q^2956, ^r^2713, ^s^711, ^t^169

#### Outcomes

Those patients with prior stroke at recruitment had a significant increase in frequencies of incident stroke (8.8% v 2.9% *p* < 0.01), myocardial infarction (10.6% v 5.4% *p* < 0.01), all NFCVE (26.4% v 11.3% *p* < 0.01), reaching ESRD (40.5% v 31.3% *p* < 0.01) and all-cause mortality (69.2% v 45% *p* < 0.01) compared to patients with no prior stroke (Table 2). The renal outcomes were different between groups. A higher proportion of patients with stroke at recruitment reached eGFR< 10 mL/min (48.9% v 29.2%, *p* < 0.000) and yet less commenced dialysis (42.2% v 61.4% *p* < 0.001). There was no significant difference between CKD progression as assessed by eGFR decline between the two groups (− 1.54 mL/min/1.73m^2^/year in stroke group vs − 1.35 mL/min/1.73m^2^/year in non-stroke group *p* = 0.53).

**Table 2:** A comparison of outcomes between patients with a history of stroke at recruitment compared with those without

Outcome	Stroke at recruitment		*P* Value (stroke at recruitment v no stroke at recruitment)
	No *N* = 2833	Yes *N* = 227	
Non-Fatal Stroke	81 (2.9%)	20 (8.8%)	0.000
Non-Fatal Myocardial infarction	154 (5.4%)	24 (10.6%)	0.001
Non-fatal cardiovascular events	319 (11.3%)	60 (26.4%)	0.000
ESRD	884 (30.6%)	90 (49.6%)	0.004
First method of RRT			
Dialysis	542 (61.3%)	37 (41.1%)	0.000
Transplant	83 (9.4%)	8 (8.9%)	0.835
eGFR < 10	259 (29.3%)	45 (50%)	0.000
All-cause mortality	1275 (45%)	157 (69.2%)	0.000
Death from cardiovascular disease^a^	174 (43.5%)	31 (57.5%)	0.048
Death from stroke^a^	19 (4.75%)	6 (1.2%)	0.052
Age at death (years)^b^	77 (70–83)	77 (71–83)	0.959
Months in study	38 (16–71)	28 (10–62)	0.001
eGFR progression slope (mL/min/1.73m^2^/year)^c^	−1.35 (−4.08, 0.70)	−1.54 (−3.77, 0.26)	0.527

Continuous variables expressed as median (interquartile range) and categorical variables presented as number (%)

Non-fatal cardiovascular events variable represents a composite of stroke, transient ischaemic attack, non-fatal myocardial infarction, coronary revascularisation including coronary artery bypass grafting and cardiac arrest

*Abbreviations*: *non-fatal cardiovascular events* Non-fatal cardiovascular event, *ESRD* End Stage Renal Disease, *eGFR* estimated glomerular filtration fate (CKD-EPI)

^a^based on cause of death data available for 457patients (403 no stroke at recruitment patients and 54 patients with stroke at recruitment), cardiovascular disease includes a composite of stroke, myocardial infarction, heart failure, peripheral vascular disease, aortic aneurysm

^b^based upon 1432 deaths

^c^based upon 2885 patients who had more than 2 creatinine measurements during study

**Fig. 2:**
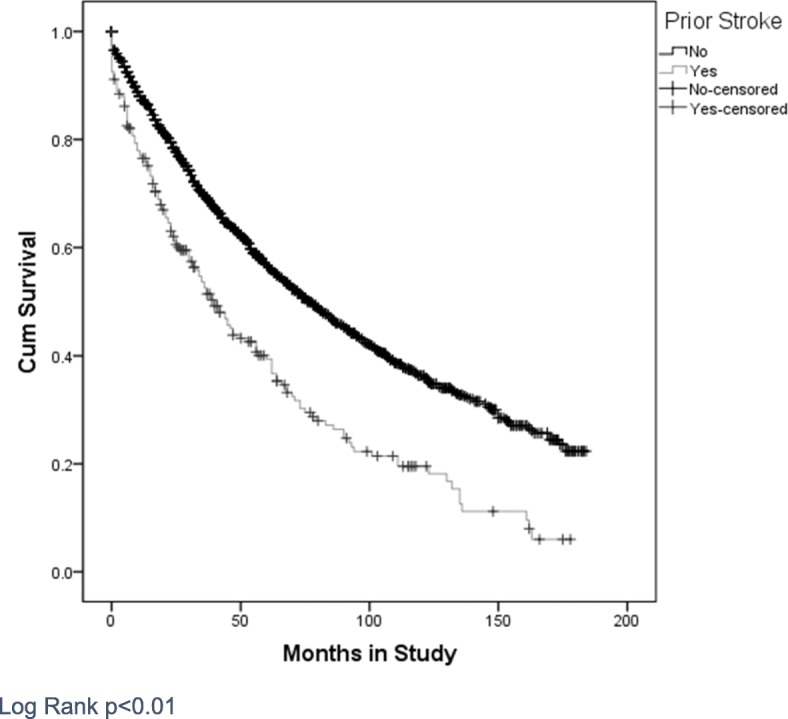
A Kaplan Meier survival curve for all-cause mortality for patients from study recruitment. Censored for follow up

Unadjusted survival analysis with the Kaplan-Meier method demonstrated worse survival in those with prior stroke (median survival 40 months vs 77 months) (Fig. 2). One, three- and five-year survival were lower in those with prior stroke compared to those without (68.3, 54.3 and 32.2% vs 86.8, 65.9 and 47.8% respectively).

Multivariable cox regression (Table 3) demonstrated that stroke prior to recruitment into the study was independently associated with mortality (HR 1.20 95%CI 1.0–1.43, *p* = 0.05), ESRD (HR 1.34 95%CI 1.06–1.69, *p* = 0.02) and future NFCVE (HR 1.54 95%CI 1.12–2.11, *p* = 0.01) after adjustment for age, gender, eGFR, diabetes, hypertension, myocardial infarction, heart failure, atrial fibrillation, smoking history and peripheral vascular disease. To account for missing data multiple imputation was performed with results similar to complete case analysis (Additional file 1: Table S2a-c). Univariate regressions are displayed in Additional file 1: Table S3. Patients with a stroke prior to study recruitment had significantly less time under study scrutiny before they reached an endpoint (28 months v 38 months *p* < 0.001). A significant interaction was found between stroke and history of MI with risk of mortality that was additive in nature (*P* = 0.004, Additional file 1: Table S4). Similarly, significant interactions were also detected between stroke and diabetes, and stroke and GFR < 30 ml/min, with future risk of mortality (*p* = 0.003 and *p* = 0.021 respectively).

**Table 3:** Multivariable Cox regression analysis: stroke at recruitment and all-cause mortality, ESRD and NFCVE

	All-cause mortality		ESRD		NFCVE	
	HR (95%CI)	*p*-value	HR (95%CI)	*p*-value	HR (95%CI)	*p*-value
Univariate model	1.79 (1.52–2.11)	0.000	1.50 (1.21–1.86)	0.000	2.73 (2.07–3.60)	0.000
Model 1	1.45 (1.23–1.71)	0.000	1.53 (1.24–1.91)	0.000	2.25 (1.70–2.97)	0.000
Model 2	1.34 (1.13–1.58)	0.001	1.38 (1.11–1.72)	0.004	2.17 (1.64–2.88)	0.000
Model 3	1.17 (0.99–1.39)	0.065	1.32 (1.05–1.65)	0.017	1.59 (1.17–2.15)	0.002
Model 4	1.20 (1.00–1.43)	0.050	1.34 (1.06–1.69)	0.016	1.54 (1.12–2.11)	0.011

Model 1: Adjusted for age, gender

Model 2: Adjusted for model 1 plus recruitment eGFR (CKD-EPI)

Model 3: Adjusted for model 2 plus diabetes, myocardial infarction, heart failure, peripheral vascular disease, hypertension and atrial fibrillation

Model 4: Adjusted for model 3 plus smoking history

### Prevalent stroke at time of dialysis commencement

Of 3060 SKS participants with comprehensive data entry, 579 commenced outpatient dialysis and 48 of these participants had suffered a stroke prior to commencing dialysis. Thirty-nine patients had suffered a stroke prior to study commencement and 9 patients had suffered a stroke during study follow up and had then subsequently commenced dialysis. The stroke group consisted of 34 (69.4%) ischaemic strokes, 7 (14.3%) haemorrhagic and 7 (14.3%) where the type of stroke could not be ascertained.

#### Baseline characteristics

In a predominantly Caucasian population, there were no significant differences between comorbidities of the stroke group and non-stroke group. The two groups were similar in age. In the prevalent stroke group there were more male patients compared with the non-stroke group (79.2% v 35.2% *p* = 0.044) (Table 4).

**Table 4:** A comparison of outcomes between patients with a history of stroke at dialysis commencement compared with those without

	Stroke at dialysis commencement		*p*-value (stroke at dialysis commencement v no stroke at dialysis commencement)
	No *N* = 531	Yes *N* = 48	
Characteristics at Dialysis Initiation			
Age	64 (51–74)	68 (56.5–73.5)	0.141
Male Gender	344 (64.8%)	38 (79.2%)	0.044
Living Alone	93 (17.5%)	8 (16.7%)	0.878
Widowed	46 (8.7%)	6 (12.5%)	0.568
Ethnic Group			
Caucasian	497 (93.6%)	46 (95.8%)	0.930
Primary renal disease			
Renovascular Disease/Hypertension	106 (20.0%)	17 (35.4%)	0.012
Diabetic kidney disease	132 (24.9%)	12 (25.0%)	0.983
Glomerulonephritis/Vasculitis	104 (19.6%)	4 (8.3%)	0.055
Pyelonephritis	30 (5.6%)	2 (4.2%)	0.667
Autosomal dominant Polycystic Kidney Disease	65 (12.2%)	5 (10.4%)	0.710
Other/Unknown	94 (17.7%)	8 (16.7%)	0.857
Smoking History	351 (74.1%)	34 (75.6%)	0.826
Diabetes	195 (36.7%)	20 (41.7%)	0.497
Heart Failure	74 (13.9%)	7 (14.6%)	0.901
Myocardial infarction	78 (14.7%)	8 (16.7%)	0.712
Peripheral vascular disease	117 (22.0%)	8 (16.7%)	0.387
Atrial fibrillation	59 (11.1%)	6 (12.5%)	0.028
Haemodialysis as first dialysis modality	347 (65.3%)	32 (66.7%)	0.854
Outcomes			
Non-Fatal Stroke	23 (4.3%)	0	
Death from stroke	4 (2.9%)	0	
Non-Fatal Myocardial infarction	58 (10.9%)	4 (8.3%)	0.578
Death from acute myocardial infarction^a^	24 (17.6%)	4 (25%)	0.498
Death from cardiovascular disease^b^	50 (36.8%)	6 (37.5%)	0.954
Newly identified Atrial Fibrillation^c^	28 (5.9%)	1 (2.4%)	0.303
Transplanted	157 (29.6%)	10 (20.8%)	0.198
Time from dialysis to transplant (months)	23 (8–38)	28 (20–37)	0.336
All-cause mortality	259 (48.8%)	31 (64.6%)	0.036
Time from dialysis to death (months)	28 (12–52)	16 (6–39)	0.093
Age at death (years)	74 (64–78)	72 (64–77)	0.648
Months of follow up from dialysis initiation^d^	25 (10–47)	20 (9–38)	0.303

Continuous variables expressed as median (interquartile range) and categorical variables presented as number (%). Between group comparisons made using chi square test for categorical variables (2 sided fishers exact test when observed outcomes < 5) and Mann-U Whitney for continuous variables. Note that cause of death data available for 152 deaths (25.6% of deaths in non-stroke group and 33.3% of deaths in prevalent stroke group, *p* = 0.925, % when in relation to death data is proportion of deaths in patients whom cause of death is known)

^a^using terms myocardial infarction, coronary artery occlusion, cardiac arrest due to coronary artery disease as cause of death

^b^using terms stroke, CVA, myocardial infarction, ruptured aneurysm, mesenteric thrombosis, left ventricular failure, cardiac dysrhythmia

^c^% as proportion of patients without previous atrial fibrillation

^d^censored at 2/3/18, death, transplant or move out of area

#### Outcomes

**Fig. 3:**
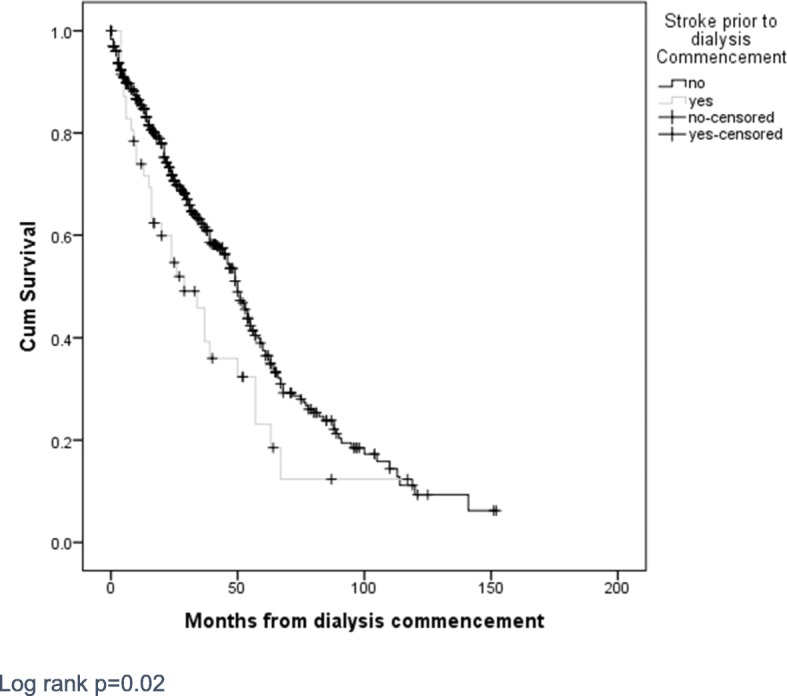
A Kaplan Meier survival curve for all-cause mortality for patients who commenced dialysis (*N* = 579). Censored for follow up

There was a significant difference in all-cause mortality between patients with prevalent stroke at dialysis commencement compared with those without (65% v 49% *p* = 0.036) (Table 4). Unadjusted survival analysis demonstrated worse survival in those with prior stroke (Fig. 3). Median survival for the prior stroke group was 29 months compared with 50 months for the non-stroke group. One, three- and five-year survival was significantly lower in those with prevalent stroke compared to the non-stroke group (76, 38 and 15% versus 85, 55 and 26% respectively).

However, prevalent stroke patients were equally likely to undergo transplantation as those without stroke and had a similar wait time for transplant whilst on dialysis. Both groups had a similar period of follow up from dialysis commencement (20 months in the stroke group v 25 months in the non-stroke group *p* = 0.303). There were no significant between-group differences in age at dialysis commencement, age at death and death from cardiovascular cause.

Multivariable cox regression (Table 5) demonstrated that stroke prior to dialysis commencement was significantly associated with mortality (HR 1.46 95%CI 1.006–2.14, *p* = 0.047) after adjustment for age, gender, diabetes, myocardial infarction, heart failure, atrial fibrillation, smoking history and peripheral vascular disease. Results of multiple imputation were similar to complete case analysis (Additional file 1: Table S5). Univariate regressions are displayed in Additional file 1: Table S6. There was no significant interaction between comorbidity variables and prior stroke with mortality outcomes (Additional file 1: Table S4).

**Table 5:** Multivariable Cox regression analysis: hazard ratio for all-cause mortality in patients who commence dialysis

	HR (95%CI)	*p*-Value
Univariate model	1.56 (1.07–2.26)	0.020
Model 1	1.49 (1.02–2.16)	0.038
Model 2	1.48 (1.02–2.16)	0.041
Model 3	1.47 (1.01–2.14)	0.047

Model 1. Adjusted for age and gender

Model 2. Adjusted for model 1 plus diabetes, myocardial infarction, heart failure, peripheral vascular disease and atrial fibrillation

Model 3. Adjusted for model 2 plus smoking history

## Discussion

In this prospective cohort study, we describe strong independent associations between stroke and the risk of major adverse clinical events in CKD. Patients who have experienced a stroke are at increased risk of mortality during the progression of CKD including at dialysis initiation. These risks were substantial and were only partially explained by adjustment of traditional CV risk factors.

Patients who suffered a stroke prior to recruitment had worse outcomes (death, ESRD and NFCVE) than those without stroke during follow up even after accounting for other known risk factors. This is a consistent with a previous Taiwanese population study (*N* = 100,353) which used coded definitions of cardiovascular events and CKD progression [18]. Our study provides additional clarity in a Caucasian population with the advantage of verification of cardiovascular events and inclusion of kidney function in the regression models.

In our study patients with a history of stroke were also less likely to commence dialysis despite having a higher rate of ESRD, reflecting that patients with previous stroke were more likely to have a non-dialysis care approach to their ESRD management. The evidence suggests that dialysis does not confer survival advantages in patients with a particularly high burden of comorbidity [19]. It is likely that those patients who had suffered a previous stroke but then commenced dialysis were those that made a more successful recovery. This may explain why renal transplantation rates were similar between those with and without prior stroke.

Patients with a prior stroke had a lower eGFR at recruitment (25.2 mL/min v 29.5 mL/min *p* < 0.01) but they were also on average 4 years older than patients without stroke. Cholesterol was lower in the prior stroke group (4.2 mmol/l v 4.5 mmol/l *p* < 0.01) most likely because of increased statin treatment (77.3% v 59.3% *p* < 0.01).

Despite good evidence that statin therapy can reduce the risk of major adverse ischaemic cardiovascular events in ND-CKD [20], and the role of statin therapy in secondary prevention of cerebrovascular disease, only 75% of prior ischaemic stroke patients were prescribed statin therapy. However, no data were available to indicate if patients had previously received statin therapy and subsequently stopped for a legitimate reason such as intolerance.

Only 70.1% of patients with an ischaemic stroke before study recruitment were prescribed antiplatelet agents, perhaps explained by different risk benefit analysis conclusions made by clinicians for secondary prevention of cerebrovascular disease in patients with CKD [21]. Inequalities of stroke care which occur in patients with concurrent renal disease may also have been a factor [22]. Data from the general population suggests that prescription of antiplatelets (97%) and lipid lowering therapy (95%) is very high in patients after ischaemic stroke [23].

Interaction analysis demonstrated a significant interaction between antiplatelets, stroke prior to recruitment and mortality. This effect was moderated but remained significant when excluding haemorrhagic, dual stroke pathology and undetermined stroke aetiologies. This finding could be demonstrating confounding by indication or it could mean that antiplatelets in CKD patients after a stroke predicts a worse outcome. This may explain why CKD patients with ischaemic stroke are not universally prescribed antiplatelets and supports Palmer et-al’s finding of uncertain benefits of antiplatelets in patients with CKD [21].

We found 8.3% of patients who commenced dialysis had suffered a previous stroke, consistent with other published data [7, 24] but lower than in the Dialysis Outcomes and Practice Patterns Study which reported a 13.7% prevalence of cerebrovascular disease. However, the latter included unverified cerebrovascular events including TIA and may therefore have over-estimated the reported prevalence [25].

The first dialysis modality choice was not proportionally different between the two groups. Two thirds of patients initially commenced haemodialysis and one third of patients commenced peritoneal dialysis irrespective of previous stroke. At present there is no clear guidance that patients with cerebrovascular disease should be offered a certain dialysis modality although recent studies suggest that peritoneal dialysis may be less harmful to the brain and reduce risk of stroke. Two longitudinal studies of prevalent dialysis patients have demonstrated that peritoneal dialysis may have less deleterious effects on patient’s cognitive function than HD [26, 27]. A Scottish national registry study also demonstrated length of time exposed to HD was independently associated with stroke whereas length of time exposed to peritoneal dialysis was not [28].

The prevalence of atrial fibrillation in the whole cohort at recruitment was 6.9% which is slightly lower than other published studies although similar methodology for atrial fibrillation data collection were used in both studies [29]. Patients did not undergo routine ECG testing so asymptomatic episodes of atrial fibrillation may have been missed.

Patients with a history of stroke who commenced dialysis had an increased risk of death after adjustments for other traditional cardiovascular risk factors. Other studies have also demonstrated that prior stroke is associated with higher mortality after dialysis commencement but without adjustment for confounding cardiovascular comorbidities [24]. In patients where cause of death was known, there was no significant difference in cardiovascular-specific death between the two groups who started dialysis. The unadjusted incidence of stroke after commencement of dialysis was 14.9 per 1000 patient years which is lower than in some published studies [6, 24] but similar to others [7]. This may be because cause of death was not available in the majority of patients, we did not include TIA as a stroke end-point and very few patients were from African American background who may have a higher risk of stroke independent of traditional risk factors [30].

It is encouraging that patients with a history of stroke and who were fit enough to commence dialysis had comparable chance of transplantation. Transplant rates and waiting times whilst undergoing dialysis were similar for both groups. Outcomes of transplantation in patients with a previous stroke are favourable and therefore clinicians should not be biased against transplant referral in patients with a prior stroke who are otherwise fit for transplantation [31].

The main strengths of this study are the large population size and the detailed characterisation of this CKD cohort, with thorough cardiovascular event assessment and long follow up duration.

A limitation of the study was the inability to validate the type of stroke in 50 patients at recruitment, 7 of whom commenced dialysis. Data on stroke severity at time of presentation or whether patients with ischaemic strokes received thrombolysis were not available. In addition, dialysis adequacy parameters were not included in regression models. Ischaemic and haemorrhagic stroke were combined and due to small numbers, analysis and outcomes were not split by the type of stroke. Furthermore, cause of death data was not available for all patients. Socioeconomic status was also not available in this dataset and is known to impact on CKD, vascular disease and mortality. Finally, this study was performed in a predominantly Caucasian population in a developed country. The South Asian population have a particularly high risk of stroke and cardiovascular disease which occurs at a younger age and is disproportionate to socio-economic or comorbid status [32]. The magnitude of the effect of stroke on patient and CKD outcomes in non-Caucasian CKD populations requires further study.

## Conclusion

In this large prospective cohort of CKD patients, a diagnosis of stroke was independently associated with several major clinical outcomes including death, reaching ESRD and suffering another non-fatal cardiovascular event. These associations highlight the importance of the brain-kidney interactions in determining patient outcomes at critical timepoints along the CKD pathway.

## Supplementary information

**Additional file 1:** **Table S1.** A table to demonstrate medication prescriptions in patients with a stroke at recruitment. **Table S2.** Multivariable Cox regression analyses: Stroke at recruitment and all-cause mortality (a), ESRD (b) and NFCVE (c). Sensitivity analysis after multiple imputation for missing data. **Table S3.** Univariate cox regression analysis for all factors collected at recruitment. **Table S4.** A table to demonstrate the interactions between variables and baseline stroke status for the three patient outcomes. Interactions are also shown for variables with prevalent stroke status at dialysis commencement and all-cause mortality. **Table S5.** Multivariable Cox regression analysis: Hazard ratio for all-cause mortality in patients who commence dialysis. Sensitivity analysis after multiple imputation for missing data. **Table S6.** Univariate cox regression analysis for death in the patients who commence dialysis.

## Data Availability

The datasets used and analyzed during the current study are available from the corresponding author on reasonable request.

## Abbreviations

CKD: Chronic kidney disease
CKD-EPI: CKD Epidemiology Collaboration
CRISIS: Chronic Renal Insufficiency Standards Implementation Study
eGFR: Estimated glomerular filtration rate
ESRD: End stage renal disease
HD: Haemodialysis
HR: Hazard ratio
ND-CKD: Non dialysis chronic kidney disease
NFCVE: Non-fatal cardiovascular event
PD: Peritoneal dialysis
RAS: Renin angiotensin system
SKS: Salford Kidney Study
TIA: Transient ischaemic attack
