# Supplementary material for: Impact of prior stroke on major clinical outcome in chronic kidney disease: the Salford kidney cohort study
Source: BMC Nephrol. 2019 Nov 27;20:432. doi: 10.1186/s12882-019-1614-5 (PMC6880597; doi:10.1186/s12882-019-1614-5)
Supplement: Supplementary file 1 — Additional file 1: Table S1. A table to demonstrate medication prescriptions in patients with a stroke at recruitment. Table S2. Multivariable Cox regression analyses: Stroke at recruitment and all-cause mortality (a), ESRD (b) and NFCVE (c). Sensitivity analysis after multiple imputation for missing data. Table S3. Univariate cox regression analysis for all factors collected at recruitment. Table S4. A table to demonstrate the interactions between variables and baseline stroke status for the three patient outcomes. Interactions are also shown for variables with prevalent stroke status at dialysis commencement and all-cause mortality. Table S5. Multivariable Cox regression analysis: Hazard ratio for all-cause mortality in patients who commence dialysis. Sensitivity analysis after multiple imputation for missing data. Table S6. Univariate cox regression analysis for death in the patients who commence dialysis. [file 12882_2019_1614_MOESM1_ESM.docx]

### Table S1

A table to demonstrate medication prescriptions in patients with a stroke at recruitment

|  | Stroke at recruitment (N=227) | | | |
| --- | --- | --- | --- | --- |
|  | Ischaemic  (N= 158) | Haemorrhagic (N=15) | Both ischaemic and haemorrhagic (N=4) | Undetermined  (N=50) |
| Prescribed anti-platelet | 110 (70.1%) | 8 (53.3%) | 3 (75%) | 37 (74%) |
| Prescribed dual antiplatelet | 17 (10.8%) | 1 (6.7%) | 0 (0%) | 2 (4%) |
| Prescribed anticoagulation | 22 (14.0%) | 0 (0%) | 0 (0%) | 7 (14%) |
| Prescribed statin | 118 (75%) | 11 (73.3%) | 2 (50%) | 41 (82%) |

### Table S2a

Multivariable Cox regression analysis: Stroke at recruitment and all-cause mortality. Sensitivity analysis after multiple imputation for missing data:

|  | HR (95%CI) | p-value |
| --- | --- | --- |
| Univariate model | 1.79 (1.67-1.91) | 0.000 |
| Model 1 | 1.45 (1.36-1.55) | 0.000 |
| Model 2 | 1.34 (1.14-1.59) | 0.001 |
| Model 3 | 1.19 (1.00-1.40) | 0.047 |
| Model 4 | 1.18 (1.00-1.40) | 0.054 |

^Model 1: Adjusted for age, gender.^

^Model 2: Adjusted for model 1 plus recruitment eGFR (CKD-EPI)^

^Model 3: Adjusted for model 2 plus diabetes, myocardial infarction, heart failure, peripheral vascular disease, hypertension and atrial fibrillation^

^Model 4: Adjusted for model 3 plus smoking history^

### Table S2b

Multivariable Cox regression analysis: Stroke at recruitment and reaching ESRD (Transplant, eGFR< 10, start dialysis). Sensitivity analysis after multiple imputation for missing data:

|  | HR (95%CI) | p-value |
| --- | --- | --- |
| Univariate model | 1.50 (1.21-1.86) | 0.000 |
| Model 1 | 1.53 (1.37-1.71) | 0.000 |
| Model 2 | 1.45 (1.16-1.80) | 0.001 |
| Model 3 | 1.35 1.07-1.71) | 0.012 |
| Model 4 | 1.35 (1.07-1.70) | 0.011 |

^Model 1: Adjusted for age, gender.^

^Model 2: Adjusted for model 1 plus recruitment eGFR (CKD-EPI)^

^Model 3: Adjusted for model 2 plus diabetes, and prior myocardial infarction, heart failure, peripheral vascular disease, hypertension or atrial fibrillation^

^Model 4: Adjusted for model 3 and smoking history^

### Table S2c

Multivariable Cox regression analysis: Stroke at recruitment and non-fatal cardiovascular events. Sensitivity analysis after multiple imputation for missing data:

|  | HR (95%CI) | p-value |
| --- | --- | --- |
| Univariate model | 2.73 (2.07-3.60) | 0.000 |
| Model 1 | 2.25 (1.95-2.59) | 0.000 |
| Model 2 | 2.16 (1.63-2.85) | 0.000 |
| Model 3 | 1.59 (1.19-2.13) | 0.002 |
| Model 4 | 1.60 (1.20-2.14) | 0.001 |

^Model 1: Adjusted for age, gender.^

^Model 2: Adjusted for model 1 plus recruitment eGFR (CKD-EPI)^

^Model 3: Adjusted for model 2 plus diabetes, and myocardial infarction, heart failure, peripheral vascular disease, hypertension or atrial fibrillation^

^Model 4: Adjusted for model 3 and smoking history^

### Table S3

Univariate cox regression analysis for all factors collected at recruitment

| Variable | HR All-cause mortality (95%CI) | p-Value | HR for ESRD (95%CI) | p-Value | HR for NFCVE (95%CI) | p-value |
| --- | --- | --- | --- | --- | --- | --- |
| Stroke | 1.79 (1.52-2.11) | 0.000 | 1.50 (1.21-1.86) | <0.01 | 2.78 (2.10-3.67) | <0.01 |
| Age | 1.05 (1.05-1.05) | <0.01 | 1.00 (0.99-1.00 | 0.04 | 1.04 (1.04-1.05) | <0.01 |
| Smoking history | 1.85 (1.62-2.12) | <0.01 | 1.30 (1.12-1.52) | <0.01 | 1.33 (1.04-1.69) | 0.02 |
| Living alone | 1.27 (1.12-1.44) | 0.000 | 0.929 (0.789-1.095) | 0.38 | 1.199 (0.937-1.535) | 0.15 |
| Hypertension | 1.73 (1.38-2.16) | <0.01 | 1.80 (1.37-2.38) | <0.01 | 1.71 (1.11-2.62) | 0.02 |
| Diabetes | 1.72 (1.55-1.91) | <0.01 | 1.37 (1.20-1.56) | <0.01 | 2.16 (1.76-2.65) | <0.01 |
| Atrial fibrillation | 1.51 (1.25-1.81) | <0.01 | 0.79 (0.59-1.06) | 0.79 | 1.89 (1.33-2.70) | <0.01 |
| Systolic BP | 1.0 (1.0-1.0) | 0.25 | 1.01 (1.01-1.02) | <0.01 | 1.01 (1.01-1.02) | <0.01 |
| Diastolic BP | 1.0 (0.99-1.0) | <0.01 | 1.01 (1.01-1.02) | <0.01 | 0.98 (0.97-0.99) | <0.01 |
| Myocardial infarction | 2.02 (1.79-2.28) | <0.01 | 1.00 (0.83-1.20) | 0.99 | 3.80 (3.08-4.70) | 0.000 |
| Heart failure | 2.17 (1.94-2.44) | <0.01 | 1.25 (1.06-1.47) | <0.01 | 2.64 (2.10-3.31) | <0.01 |
| Peripheral vascular disease | 1.83 (1.61-2.07) | <0.01 | 1.40 (1.19-1.66) | <0.01 | 1.80 (1.39-2.33) | <0.01 |
| RAS blockade | 0.71 (0.64-0.79) | <0.01 | 0.88 (0.77-1.00) | 0.05 | 0.69 0.56-0.85) | <0.01 |
| Statin use | 1.19 (1.07-1.33) | <0.01 | 1.12 (0.98-1.27) | 0.10 | 1.82 (1.45-2.29) | <0.01 |
| Urine PCR (g/mol) | 1.00 (1.00-1.00) | <0.01 | 1.00 (1.00-1.00) | <0.01 | 1.00 (1.00-1.00) | 0.1 |
| eGFR (mL/min/1.74m^2^) | 0.95 (0.95 -0.96) | <0.01 | 0.90 (0.89-0.91) | <0.01 | 0.97 (0.96-0.98 | <0.01 |

^Heart failure data missing in 115 cases, hypertension 18 cases, Systolic and diastolic blood pressure measurements in 475 cases, UPCR 169, Smoking data in 204 cases, statin use 62 cases, RAS use 61 cases. Abbreviations: RAS=Renin aldosterone blockade, PCR=protein creatinine ratio, eGFR=estimated glomerular filtration rate (CKD-EPI)^

Table S4

Two-way interaction tests between baseline cardiovascular risk factors or protective medications and mortality, end-stage renal disease or non-fatal cardiovascular events

| Baseline factor | Significance of interaction of prior stroke with baseline factors stratified by outcomes in CKD cohort (n=3060) | | | Significance of interaction of prior stroke with baseline factors and all-cause mortality in dialysis cohort (n=579) |
| --- | --- | --- | --- | --- |
|  | All-cause mortality | ESRD | Future NFCVE |  |
| Myocardial infarction | 0.004 | 0.211 | 0.734 | 0.903 |
| Heart Failure | 0.171 | 0.205 | 0.159 | 0.755 |
| eGFR<30 | 0.485 | 0.937 | 0.021 | Not applicable |
| Peripheral vascular disease | 0.559 | 0.786 | 0.062 | 0.435 |
| Atrial fibrillation | 0.977 | 0.912 | 0.319 | 0.455 |
| Hypertension | 0.326 | 0.489 | 0.256 | 0.905 |
| Diabetes | 0.268 | 0.248 | 0.003 | 0.731 |
| ACE/ARB therapy | 0.788 | 0.156 | 0.379 | * |
| Antiplatelets | 0.005^a^ | 0.192^b^ | 0.013^c^ | * |
| Statin therapy | 0.892 | 0.382 | 0.015 | * |

^*Medications for patients at the point of dialysis commencement are not routinely collected as part of the Salford Kidney Study. After excluding haemorrhagic, mixed and unknown stroke aetiologies (157 baseline strokes in 2930 patients) p values for two-way interaction were a=0.050, b=0.196 c=0.002^.

Table S5

Multivariable Cox regression analysis: Hazard ratio for all-cause mortality in patients who commence dialysis. Sensitivity analysis after multiple imputation for missing data:

|  | HR (95%CI) | p-Value |
| --- | --- | --- |
| Univariate model | 1.56 (1.07-2.26) | 0.020 |
| Model 1 | 1.49 (1.02-2.16) | 0.038 |
| Model 2 | 1.48 (1.02-2.16) | 0.041 |
| Model 3 | 1.48 (1.01-2.16) | 0.042 |

^Model 1. Adjusted for age and gender^

^Model 2. Adjusted for model 1 plus diabetes, myocardial infarction, heart failure, peripheral vascular disease and atrial fibrillation^

^Model 3. Adjusted for model 2 plus smoking history^

###

### Table S6

Univariate cox regression analysis for death in the patients who commence dialysis

| Variable | HR | 95% CI | p-value |
| --- | --- | --- | --- |
| Stroke | 1.559 | 1.073-2.264 | 0.020 |
| Age (per year) | 1.040 | 1.029-1.051 | 0.000 |
| Peripheral vascular disease | 1.031 | 0.789-1.346 | 0.824 |
| Myocardial infarction | 1.185 | 0.891-1.594 | 0.263 |
| Diabetes | 1.384 | 1.097-1.746 | 0.006 |
| Heart failure | 1.426 | 1.076-1.891 | 0.014 |
| Male | 0.897 | 0.698-1.153 | 0.395 |
| Atrial fibrillation | 0.823 | 0.570-1.189 | 0.299 |
| Smoking history | 1.464 | 1.053-2.036 | 0.023 |
| Living alone | 1.048 | 0.782-1.404 | 0.753 |
| Widowed | 1.063 | 0.949-1.192 | 0.289 |
